# Feasibility and antihypertensive effect of replacing regular salt with mineral salt -rich in magnesium and potassium- in subjects with mildly elevated blood pressure

**DOI:** 10.1186/1475-2891-10-88

**Published:** 2011-09-02

**Authors:** Essi S Sarkkinen, Mika J Kastarinen, Tarja H Niskanen, Pia H Karjalainen, Taisa M Venäläinen, Jay K Udani, Leo K Niskanen

**Affiliations:** 1 Oy Foodfiles Ltd, CRO in the field of nutrition, Neulaniementie 2 L 6, 70210 Kuopio, Finland; 2 University of Eastern Finland, Institute of Public Health and Clinical Nutrition, Department of Clinical Nutrition, Food and Health Research Centre, Kuopio, Finland; 3 Department of Medicine, Central Hospital Central Finland, Jyväskylä, Finland, and University of Eastern Finland, Faculty of Health Sciences, School of Medicine, Kuopio, Finland; 4 Medicus Research LLC/UCLA School of Medicine, Northridge, California, USA

**Keywords:** blood pressure, hypertension, sodium reduction, mineral salt, low-sodium, high-potassium and high-magnesium salt

## Abstract

**Background:**

High salt intake is linked to hypertension whereas a restriction of dietary salt lowers blood pressure (BP). Substituting potassium and/or magnesium salts for sodium chloride (NaCl) may enhance the feasibility of salt restriction and lower blood pressure beyond the sodium reduction alone. The aim of this study was to determine the feasibility and effect on blood pressure of replacing NaCl (Regular salt) with a novel mineral salt [50% sodium chloride and rich in potassium chloride (25%), magnesium ammonium potassium chloride, hydrate (25%)] (Smart Salt).

**Methods:**

A randomized, double-blind, placebo-controlled study was conducted with an intervention period of 8-weeks in subjects (n = 45) with systolic (S)BP 130-159 mmHg and/or diastolic (D)BP 85-99 mmHg. During the intervention period, subjects consumed processed foods salted with either NaCl or Smart Salt. The primary endpoint was the change in SBP. Secondary endpoints were changes in DBP, daily urine excretion of sodium (24-h dU-Na), potassium (dU-K) and magnesium (dU-Mg).

**Results:**

24-h dU-Na decreased significantly in the Smart Salt group (-29.8 mmol; p = 0.012) and remained unchanged in the control group: resulting in a 3.3 g difference in NaCl intake between the groups. Replacement of NaCl with Smart Salt resulted in a significant reduction in SBP over 8 weeks (-7.5 mmHg; p = 0.016). SBP increased (+3.8 mmHg, p = 0.072) slightly in the Regular salt group. The difference in the change of SBP between study groups was significant (p < 0.002).

**Conclusions:**

The substitution of Smart Salt for Regular salt in subjects with high normal or mildly elevated BP resulted in a significant reduction in their daily sodium intake as well as a reduction in SBP.

**Trial Registration:**

ISRCTN: ISRCTN01739816

## Background

Hypertension is a major risk factor for cardiovascular diseases (CVD) as well as for renal diseases [1]. Reducing salt intake lowers blood pressure and consequently lowers the risk of cardiovascular disease. The observed decline in the prevalence of hypertension has levelled off [2,3]. Therefore, there is an evident need for public health measures aiming at more effective lifestyle modifications connected to high BP including the reduction of sodium intake [4-7].

Meta-analyses show that a daily sodium reduction of at least 53 mmol (3.1 g/day as sodium chloride (NaCl)) can lead to a reduction of 4-5 mmHg in systolic BP (SBP) and 2-3 mmHg in diastolic BP (DBP) in hypertensive subjects [8-15].

The supplementation of potassium and magnesium to a low-sodium diet may enhance the antihypertensive effect of a low-sodium diet, and thus be useful in treating hypertension [16-18]. Potassium has a natriuretic effect, promoting the excretion of sodium salts in the urine [17]. Intake of magnesium may also support BP lowering by reducing vascular resistance [19].

Earlier studies have demonstrated the antihypertensive effect of replacing normal salt with low-sodium, potassium and magnesium containing mineral salt [20-26].

Accomplishing the sodium restriction has been challenging and target levels are not achieved in everyday life. It has been estimated that cutting down salt concentrations in processed foods could be the most feasible way to reduce salt intake at the population level [7,27,28].

Only preliminary data is available on mineral salt containing magnesium over 2% of total weight. The goal of this study was to test the feasibility and effect of a new mineral salt (Smart Salt) on blood pressure, which is low in sodium and high in potassium and magnesium. The hypothesis of the study was that the replacement of Smart Salt for a regular salt covering at least 50% of dietary sodium sources in a diet would lead to a 3 to 5 g reduction in sodium intake and consequently a lower SBP.

## Methods

### Subjects

Subjects were recruited via announcements in the local newspapers and from the volunteer register of Oy Foodfiles Ltd in the Kuopio area, Eastern Finland. A written consent form was obtained from each subject. Subjects were eligible if they were between 25-75 years old, with their SBP in the range of 130-159 mmHg and/or DBP in the range of 85-99 mmHg, BMI between 23 and 40 kg/m^2 ^and a stable body weight. Subjects were excluded if they were taking antihypertensive drugs, non-steroidal anti-inflammatory agents, cyclosporine or tacrolimus. They were also excluded if they had secondary hypertension, diabetes (type 1 or 2), a history of active heart disease or cancer, abnormal electrolytes, proteinuria, abnormal liver, kidney or thyroid function. Subjects were also excluded if they were currently on a low-salt diet (six or less points in the salt intake test by the Finnish Heart Association, Helsinki). Subjects with alcohol abuse (> 14 units per week) or drug abuse were excluded. Pregnant and lactating mothers were also excluded.

### Study Design

**Figure 1:**
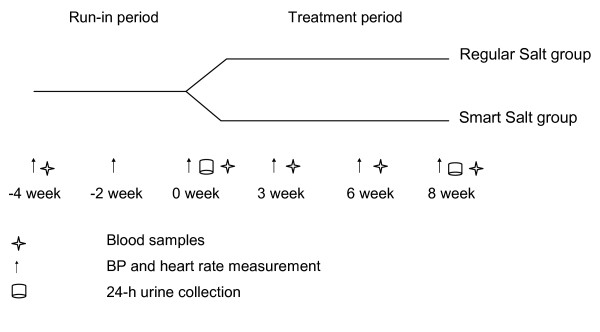
**Study Design**. This randomized, double-blind, placebo-controlled, parallel study was comprised of a run-in period and a treatment period.

This was a randomized, double-blind, placebo-controlled, parallel study (Figure 1). The study consisted of six study visits, beginning with 2 visits during the 3-5 week run-in period on a habitual diet. At the third visit (week 0), the subjects were randomized into one of the two study groups: test (Smart Salt group) or control (Regular salt group) for 8 weeks. The study was conducted according to the guidelines laid down in the Declaration of Helsinki. The Ethics Committee of the Hospital District of Northern Savo approved the study protocol.

### Study products and diets

The test product was Smart Salt^® ^SMS50 supplied by Smart Salt^® ^Inc (California USA). Smart Salt^® ^contained 50% sodium chloride (NaCl), 25% potassium chloride (KCl) and 25% magnesal; magnesium ammonium potassium chloride, hydrate [Mg_4_K(NH_4_)_3_Cl_12_·24H_2_O]. The control was a regular salt (sodium chloride, NaCl) (Akzo Nobel Salt, Netherlands).

During the treatment period, the main food sources of salt were either salted as normal or with Smart Salt^®^, depending on the study group. Test foods were industrially processed main dishes (casseroles, soups, pastas, pizza and minced meat dishes), bread (70% rye bread and 30% multigrain), frankfurters sausage/cold cuts and Edam cheese. Salt used for cooking and baking as well as table salt was either regular salt or Smart Salt^®^, dependent on group.

The daily amount of the test foods in the study diet was based on national dietary data in Finland, namely the FinDiet 2007 study. In the test group, the NaCl reduction was designed to be 3.1 to 5.6 grams (1.2 to 2.2 grams Na^+^) depending on the energy intake and habitual diet of the study subject. The goal was to replace approximately 60% of the regular sources of sodium with Smart Salt products in the intervention group. The daily sodium intake in the Regular Salt group was designed to stay at the same level as typical for that individual. The amounts of Smart Salt and Regular Salt in recipes of test foods were the same. The analyzed concentration of sodium (expressed as NaCl) in the test foods varied between 0.38-1.41% in Smart Salt foods and 0.64-2.03% in Regular salt foods depending on the food matrix.

All study subjects were told by a nutritionist to refrain from salt-rich products (such as salty snacks, soy sauce, olives, salt-rich cheeses, stock cubes, salty and smoked fish etc.). The use of products containing bioactive peptides (like Evolus^®^), salts other than the test salts, licorice (*Radix glycyrrhizae*), ammonium chloride products and any food supplements that might affect BP were also prohibited. Study subjects could freely consume liquid dairy products, vegetables, fruits and berries in addition to the study foods.

#### Blood pressure and heart rate measurements

BP and heart rate were measured using an automatic sphygmomanometer (Omron M4-I, fully automatic BP monitor, Omron Matsusaka Co, Ltd, Japan) following 10 minutes rest in a sitting position. BP was measured three times with intervals of at least two minutes, between the hours of 7:00 am and 12:00 noon. The mean of the last two BP measurements was used as the result. BP was measured using the non-dominant arm with the exception of the first study visit during which BP was measured using both arms. If the BP in the two arms differed in SBP or DBP by more than 10 mmHg, the arm with the higher reading was used for all subsequent measurements. Volunteers were not told the results of their BP measurements during the study and the study nurse was unaware of the treatment allocations.

#### Weight and height measurements

Body weight was measured using a calibrated digital scale (Scale Seca 704, Medical scales and measuring systems Seca GmbH & Co, Germany). Height was measured with a *Seca *telescoping measuring rod type 221 (Vogel & Halke GmbH & Co, Germany) to the nearest crossed half a centimeter at the first study visit (-4 wk). Body mass index (BMI) was calculated with the equation: weight (kg)/height (m)^2^.

#### 24-hour urine collection

The subjects completed 24 hour urine collections twice during the study; before the intervention period (-1 day) and once at end of the intervention period (+8 wk). Urine collections were required to account for a minimum of 20 hours and a maximum of 28 hours, with the amount lost being ≤ 10% of total urine. Urine samples were analyzed for sodium, potassium, magnesium and creatinine at the ISLAB laboratories, Kuopio. Urinary creatinine was analyzed using an enzymatic method. Urinary potassium and sodium were measured using an ion-specific electrode (ISE). Magnesium was measured using atomic absorption spectrometry. The completeness of urine excretion was checked in the present study by calculating the sodium excretion in relation to 24-hour creatinine excretion.

#### Blood Samples

Blood samples were taken after a 10 to 12 hour overnight fasting period and after BP had been measured. Blood samples were analyzed using standardized methods of hematology and clinical chemistry at the ISLAB laboratories, Kuopio. Plasma sodium and potassium were analyzed by ion-specific electrode and plasma magnesium was analyzed using atomic absorption spectrometry.

#### Study Diaries

The compliance of the test protocol was assured using individual diaries in which the use of test products was recorded. The subjects also recorded in their daily diary their weight, any possible adverse effects, changes in lifestyle and medications during the study.

#### Data management and statistical analysis

Data management and analyses were performed using SPSS (version 17.0, SPSS Inc, Chicago, USA). The results are presented as means and standard deviations. The normal distribution of variables was checked using the Shapiro-Wilk test. The equality of variances among the study groups was tested using the Levene test. The equality of variance-covariance matrices across cells was tested using the Box's M test. The general linear model (GLM) for repeated measures was used to test between-groups and with-in group differences using repeated continuous variables. In the event of a significant time/group interaction, independent sample t-tests were conducted using the Bonferroni correction in among-group analyses and comparisons within groups were conducted using paired sample t-tests. For the continuous variables, which were not normally distributed even after logarithmic transformation, the Mann-Whitney test (among groups) and Wilcoxon test or Friedman test (with-in group) were conducted with Bonferroni correction when applicable. P-values less than 0.05 were regarded as statistically significant.

## Results

### Subjects

Telephone screening was performed on 72 volunteer male and 96 female volunteers. Of those screened by telephone fifty (50) eligible subjects (24 men and 26 women) were randomized into the study. Forty five (45) subjects (22 in the Smart Salt group and 23 in the Regular salt group) completed the study and were included in the data analysis (90% completion rate). At baseline, there were no significant differences between the study groups in mean BP, BMI or any of the routine laboratory measurements (Table 1). The most typical energy level in the Smart Salt group was 2200 kcal and in the Regular Salt group 2000 kcal respectively.

**Table 1:** Characteristics of the study subjects.

	Smart Salt^®^ (n = 22)	Regular salt (n = 23)
**Female/Male (n)**	13/9	9/14
**Age (years)**	57 (12)	54 (11)
**Pre-trial blood pressure (mmHg)^1^**		
**Systolic**	141 (8)	138 (9)
**Diastolic**	87 (6)	88 (7)
**Pre-trial body weight (kg)**	77 (12)	85 (17)
**Body weight (kg) (Visit 3)**	77 (12)	85 (16)
**Body weight (kg) (Visit 6)**	77 (13)	84 (16)
**Pre-trial body mass index (kg/m^2^)**	28 (3)	28 (3)
**Body mass index (kg/m^2^) (Visit 3)**	28 (3)	28 (3)
**Body mass index (kg/m^2^) (Visit 6)**	28 (3)	28 (3)
**Smokers (n)**	3	3
**Alcohol users (n)**	18	21
**Subjects having regular^2 ^exercise (n)**	19	19
**Pre-trial consumption of salt (points)^3^**	11.7 (2.5)	11.9 (1.4)
**Subjects using vitamin/mineral supplements before intervention (n)**	13	10
**Subjects using vitamin/mineral supplements during intervention (n)**	8	7
**Subjects using other food supplements (n)**	7	8

^1 ^mean of two measurements (Visit 1, -4 weeks and Visit 2, -2 weeks)

^2 ^exercising at least 2-3 times/week for 30 minutes or more

^3 ^according to the salt intake test (Finnish Heart Association, Helsinki)

P-values < 0.05 are considered as statistically significant

### Consumption of test products

The mean daily consumption of test products was 95% in the Smart Salt group and 88% in the Regular salt group, according to the subject's diaries. During the intervention period, the Smart Salt group used an average of 1.9 (± 2.2) grams of table salt per day and the Regular salt group used an average of 1.3 (± 1.0) grams per day (NS).

### Dietary sodium chloride intake

The sodium chloride (NaCl) intake calculated from dU-Na and product diaries are

presented in Table 2. At the end of the 8 week intervention, mean daily NaCl intake calculated from dU-Na, was significantly less in the Smart Salt group compared to that of the Regular salt group (-3.3 g; p < 0.002). In the Smart Salt group, daily NaCl intake, decreased by 2.0 grams compared to baseline (p = 0.012) while the change from baseline in the Regular salt group was insignificant. The difference in absolute change of NaCl intake between study groups was of borderline statistical significance (p = 0.05) whereas the percentage change between study groups was statistically significant (net difference -21.6%; p = 0.033) (Table 2).

**Table 2:** Daily salt intake (as NaCl) during intervention.

	Smart Salt^®^ (n = 22) Mean (SD)	Regular salt (n = 23) Mean (SD)	p-value
**Daily NaCl intake during intervention (calculated from product diaries) (grams)**	5.3 (1.6)	8.5 (2.0)	p < 0.001^1^
**Daily NaCl intake calculated from dU-Na (grams)**			
Visit 3 (-1 day)	8.8 (3.2)	10.2 (2.9)	NS^2^
Visit 6 (+8 weeks)	6.8 (3.3)	10.1 (3.1)	p < 0.002^2^
**p-value^3^**	p = 0.012	NS	
**Changes in daily NaCl intake during intervention calculated from dU-Na**			
grams	-2.0 (3.6)	-0.1 (2.8)	NS (p = 0.050)^4^
%	-18.5 (28.8)	3.1 (34.7)	p = 0.033^1^
**Changes in daily NaCl intake during intervention calculated from baseline dU-Na and NaCl intake calculated from diaries**			
grams	-3.5 (2.9)	-1.7 (3.7)	NS^1^
%	-35.1 (21.6)	-7.8 (40.0)	p = 0.007^1^

^1 ^Indicates the significance of difference between the groups analyzed with Mann-Whitney test.

^2 ^Indicates the significance of difference between the groups analyzed with Mann-Whitney test with Bonferroni corrections.

^3 ^Indicates the significance of difference within the group analyzed with Wilcoxon test with Bonferroni corrections.

^4 ^Indicates the significance of difference between the groups analyzed with independent samples t-test.

P-values < 0.05 are considered as statistically significant.

NS = non-significant

The mean daily NaCl intake as calculated using the product diaries was 3.2 grams less in the Smart Salt group compared to the Regular salt group during the intervention period (p < 0.001). Daily NaCl intake decreased on average by 3.5 grams in the Smart Salt group and by 1.7 g in the Regular salt group in comparison to baseline dU-Na data.

### Blood pressure

**Figure 2:**
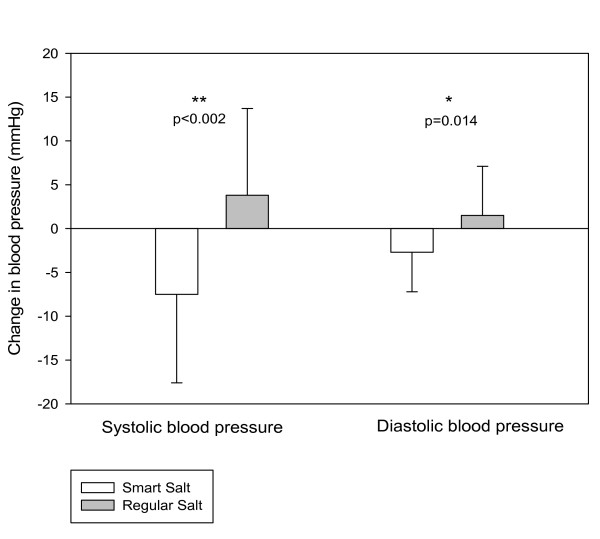
**The change in systolic (SBP) and diastolic blood pressure (DBP) are illustrated as means ± standard deviations**. P-values indicate the statistical significance for difference between the groups.

The changes in SBP and DBP from baseline were significantly different between the study groups (p < 0.002 and p = 0.014, respectively). There was a decrease in both SBP and DBP in the Smart Salt group over the 8 week intervention period (Figure 2). The mean SBP decreased by 7.5 ± 10.1 mmHg and the DBP decreased by 2.7 ± 4.5 mmHg. In contrast, there was a slight increase in SBP and DBP in the Regular salt group (+3.8 and +1.5 mmHg, respectively).

There was a significant difference in mean SBP between the study groups at week 3 (p = 0.028) and a nearly significant difference (p = 0.076) at the end of intervention (Table 3). The mean values of SBP and DBP in the Smart Salt group vs Regular Salt group are shown in Table 3. Also the mean DBP tended to be lower for the Smart Salt group compared to the Regular salt group, but the differences were not statistically significant (Table 3).

**Table 3:** Systolic and diastolic blood pressure during the intervention period.

	Smart Salt^®^ (n = 22)	Regular salt (n = 23)	p-value	p-value
**Systolic blood pressure (mmHg)**				p < 0.001^1^
Visit 3 (0 week)	140 (13)	134 (9)	NS^3^	
Visit 4 (+3 weeks)	133 (8)^a^	140 (9)	p = 0.028^3^	
Visit 5 (+6 weeks)	132 (10)^b^	139 (10)	NS^3^	
Visit 6 (+8 weeks)	132 (7)^c^	138 (9)	NS (p = 0.076)^3^	
**p-value^1 ^**(p < 0.001)				
**p-value^2^**	p = 0.016	NS (p = 0.072)		
**Diastolic blood pressure (mmHg)**				p = 0.007^1^
Visit 3 (0 week)	89 (8)	88 (7)	NS^3^	
Visit 4 (+3 weeks)	86 (6)	90 (6)	NS^3^	
Visit 5 (+6 weeks)	86 (6)	91 (8)	NS^3^	
Visit 6 (+8 weeks)	86 (7)	90 (7)	NS^3 ^	
**p-value^1 ^**(p = 0.007)				
**p-value^2^**	NS (p = 0.094)	NS		

^1 ^Indicates the significance of the time*group interaction analyzed with the GLM for repeated measures.

^2 ^Indicates the significance of difference within the groups analyzed with the GLM for repeated measures with Bonferroni corrections.

^3 ^Indicates the significance of difference between the groups analyzed with the independent samples t-test with Bonferroni corrections.

Letters indicate significance of within-group difference compared to baseline (0 wk) analyzed with paired t-test with Bonferroni corrections.

^a ^p = 0.081

^b ^p = 0.024

^c ^p = 0.006

P-values < 0.05 are considered as statistically significant.

NS = non-significant

### 24-hour urinary sodium, potassium, magnesium and creatinine

In the Smart Salt group there was a decrease in dU-Na during 8 weeks compared to baseline (-29.8 mmol; p = 0.012), whereas there was no significant change over time in the Regular salt group. At 8 week mean dU-Na in Smart Salt group was significantly less than that of the Regular Salt group (-50 mmol, p < 0.002) (Table 4). The difference in absolute change of dU-Na between study groups was of borderline statistical significance (net difference 28.8 mmol; p = 0.050) (Table 4). However, in percentage terms dU-Na decreased significantly (p = 0.033) more in Smart Salt group (-18.5 ± 28.8%) compared to a slight increase in Regular Salt group (+3.0 ± 34.7%). The between group difference remained statistically significant when dU-Na values were adjusted with dU-Crea values (p = 0.004).

**Table 4:** Total urinary sodium (du-Na), potassium (dU-K), magnesium (dU-Mg) and creatinine (dU-Crea) excretion in 24-h period before (-1 day) and during (+8 wk) the intervention.

	Smart Salt (n = 22)	Regular salt (n = 23)	p-value	p-value
**dU- Na (mmol)**				
Visit 3 (-1 day)	130 (47)	151 (44)	NS^1^	
Visit 6 (+8 weeks)	100 (49)	150 (46)	p < 0.002^1^	
**p-value**	p = 0.012^2^	NS^2^		
**Change: 0 wk vs +8 wk**	-29.8 (53.3)	-0.9 (42.0)	NS (p = 0.050)^3^	
**dU-K (mmol)**				p = 0.003^4^
Visit 3 (-1 day)	79 (31)	82 (22)	NS^5^	
Visit 6 (+8 weeks)	95 (32)	66 (22)	p = 0.002^5^	
**p-value^3 ^**(p = 0.003)				
**p-value**	NS^6^	p = 0.034^6^		
**Change: 0 wk vs +8 wk**	16.0 (37.4)	-15.7 (29.0)	p < 0.001^7^	
**dU-Mg (mmol)**			NS^8^	NS^4^
Visit 3 (-1 day)	4.54 (1.61)	4.67 (1.24)		
Visit 6 (+8 weeks)	5.14 (1.97)	4.46 (1.67)		
**p-value**^9 ^(NS)				
**Change: 0 wk vs +8 wk**	0.60 (1.82)	-0.21 (1.31)	p = 0.028^7^	
**dU-Crea (mmol)**				
Visit 3 (-1 day)	12 (4)	14 (4)	NS^1^	
Visit 6 (+8 weeks)	11 (4)	12 (4)	NS^1^	
**p-value**	NS^2^	p = 0.030^2^		
**dU-Na/dUCrea**				
Visit 3 (-1 day)	11.5 (4.1)	11.9 (3.8)	NS^1^	
Visit 6 (+8 weeks)	11.7 (12.7)	12.7 (4.0)	p = 0.004^1^	
**p-value**	NS^2^	NS^2^		

Mean (SD).

^1 ^Indicates the significance of difference between the groups analyzed with the Mann-Whitney test with Bonferroni corrections.

^2 ^Indicates the significance of difference within the group analyzed with the Wilcoxon test with Bonferroni corrections.

^3 ^Indicates the significance of difference between the groups analyzed with the independent samples t-test.

^4 ^Indicates the significance of the time*group interaction analyzed with the GLM for repeated measures.

^5 ^Indicates the significance of difference between the groups analyzed with the independent samples t-test with Bonferroni corrections.

^6 ^Indicates the significance of difference within the group analyzed with the paired samples t-test with Bonferroni corrections.

^7 ^Indicates the significance of difference between the groups analyzed with the Mann-Whitney test.

^8 ^Indicates the significance of difference between the groups analyzed with the GLM for repeated measures.

^9 ^Indicates the significance of difference within the groups analyzed with the GLM for repeated measures.

P-values < 0.05 are considered as significant.

NS = non-significant

Following 8 weeks of treatment, dU-K in the Smart Salt group increased by 16.0 mmol (a non significant change) and decreased in the Regular salt group (-15.7 mmol; p = 0.034) (Table 4). There was a significant difference (p = 0.002) in the mean of dU-K between the study groups at end of intervention (Table 4). The change in dU-K (0 vs 8 wk) differed also in absolute (Table 4 p < 0.001) and percentage terms (+30.9 ± 46.1 vs -14.2, ± 36.4; p = 0.001) between the study groups.

There were no significant differences in the mean values of dU-Mg between nor within the study groups. In Smart Salt group, dU-Mg tended to increase (+0.60 mmol) following 8 weeks intervention and in the Regular salt group, dU-Mg tended to decrease (-0.21 mmol) (Table 4). However, these absolute changes in dU-Mg (Table 4, p = 0.028) and percentage changes in dU-Mg (+20.2 ± 46.4 in Smart Salt group vs -4.4 ± 27.6% in Regular Salt group; p = 0.021) differed between the study groups significantly.

Plasma sodium, magnesium and creatinine concentrations remained stable during intervention and there were no significant differences between the study groups. Within the Regular salt group, plasma potassium concentrations decreased over the 8-week intervention (0.2 mmol/l; p = 0.024).

### Adverse events

During the intervention period, there were 7 reports of respiratory symptoms (Smart Salt group n = 5/Regular salt group n = 2), 17 reports of abdominal/intestinal symptoms (12/5) and 3 reports of cardiovascular symptoms (1/2). None of the adverse events were deemed to be related to the test salts.

## Discussion

Substitution of Smart Salt for Regular Salt in processed foods was a feasible measure that enabled the volunteers to achieve the recommended salt intake level of 5-6 grams per day. Previous studies have reported that the daily NaCl reduction should be at least 3.1 g to achieve a reduction of 4 to 5 mmHg in SBP and 2 to 3 mmHg in DBP in hypertensive subjects [13]. In this study, a reduction of only 2.0 grams (measured by dU-Na) to 3.2 grams (measured by product diaries) resulted in a marked decrease of mean SBP (-7.5 mmHg) in the Smart Salt group in contrast to a slight increase of mean SBP (+3.8 mmHg) in the control group.

At baseline, daily sodium urinary excretion and dietary NaCl intake were at typical (or slightly lower) levels consumed in western populations [29,30] The food diaries indicated that those in the Smart Salt group had their daily intake of sodium (as NaCl) reduced by 3.5 g compared to the Regular salt group. Also the difference in urinary sodium excretion between the groups at the end of intervention indicated a 3.3 g difference in mean sodium intake.

The consumption of Smart Salt mineral salt produced a stronger effect on BP than could be expected based on observed sodium excretion and previous pure sodium restriction studies [8-15]. The results reported in this paper indirectly suggest that potassium and magnesium could potentiate the effect of sodium restriction on reducing BP. This effect is in line with earlier clinical trials with mineral salts [16,20-26].

An increase of urinary potassium excretion of 63 mmol/24 hour has been associated with a decrease in supine SBP of 5.9 mmHg and in DBP of 3.4 mmHg [31]. According to Whelton et al. [17], supplementation with 2 g potassium decreases blood pressure an average of 2 to 3 mmHg. In our study, the net change in potassium excretion was about 30 mmol/24 hours, predicting a 3 mmHg reduction in SBP. The sodium to potassium ratio seems to be more important in potentiating the effect on BP than individual mineral modifications [32]. It has been postulated that the effect of potassium supplementation is reduced by half if simultaneous daily sodium intake is under 3.2 g (8 g NaCl) and can be increased by double if sodium intake is over 3.8 g (9.5 g NaCl). Interestingly our result indirectly indicates that potassium substitution could be useful even at the recommended 5-6 g NaCl intake.

The high efficacy of mineral salts used in lowering BP might also be partly explained by the increase in magnesium intake. Magnesium supplementation has little direct effect on BP, especially in hypertensive subjects [32-37]. However magnesium may have an effect on BP through interactions with sodium and potassium [20,38]. Magnesium concentration was exceptionally high in the tested mineral salt (2.3% of total weight).

It should noted that the novel mineral enrichment used in the processed foods was likely to enhance the good compliance to sodium restriction throughout the study in the Smart Salt group. The taste profile of test products was good and products could not be differentiated from each other. It can be assumed that similar sodium restriction could not be easily achieved by simple salt restriction advice only. The self-reported compliance with the use of test foods and salts was good and biochemical data support this view.

Importantly lifestyle, weight and possible medications remained stable during the intervention. Thus the results could be principally ascribed to the intervention with Smart Salt low-sodium, high-potassium, high-magnesium mineral salt. Since the sample size in this proof-of concept study was relatively small, results will need to be repeated and verified with a larger sample size.

Salt substitution in processed foods and table salt may have a large impact on cardiovascular health at population level. Recently published analysis demonstrated that reducing salt by 3 g per day is projected to reduce the annual number of new cases of CHD by 60 000 to 120 000 in USA alone [7].

In conclusion, the use of Smart Salt in processed foods helped subjects to bring their sodium intake in line with the recommended levels of 2.3 g/day (5.75 g as NaCl) [5]. Additionally, this study indicates that replacing regular salt with a mineral salt low in sodium, high in potassium and high in magnesium may be a feasible way to potentiate antihypertensive effect in subjects with mild hypertension.

## Competing interests

The authors declare that they have no competing interests.

## Authors' contributions

ESS, MJK, THN, PHK and LKN contributed to the design of the study, THN and TMV recruited the subjects and carried out the practical aspects of the study; THN conducted the statistical analyses and created the graphs, ESS and JKU wrote the first draft of the manuscript in collaboration; ESS, THN, MJK, LKN, JKU and PHK critically revised the manuscript. All authors have read and approved the final manuscript.
